# Impact of periodontitis on quality of life among subjects with rheumatoid arthritis: a cross sectional study

**DOI:** 10.1186/s12903-020-01275-4

**Published:** 2020-11-23

**Authors:** Philip Sheng Hui Han, Roslan Saub, Nor Adinar Baharuddin, Sargunan Sockalingam, Peter Mark Bartold, Rathna Devi Vaithilingam

**Affiliations:** 1 Department of Restorative Dentistry, Faculty of Dentistry, University of Malaya, 50603 Kuala Lumpur, Malaysia; 2 Department of Community Oral Health and Clinical Prevention, Faculty of Dentistry, University of Malaya, Kuala Lumpur, Malaysia; 3 Department of Rheumatology, Faculty of Medicine, University of Malaya, Kuala Lumpur, Malaysia; 4 Department of Dentistry, University of Adelaide, Adelaide, Australia

**Keywords:** Periodontitis, Rheumatoid arthritis, Oral health related quality of life, Health related quality of life

## Abstract

**Background:**

This study aimed to assess the impact of periodontitis (PD) on the health related quality of life (HRQoL) and oral health related QoL (OHRQoL) of subjects with rheumatoid arthritis (RA) and PD.

**Methods:**

Subjects from dental and RA clinics were screened. Complete periodontal examinations were performed. Subjects were divided into 4 groups: RA-PD, RA, PD and healthy controls (HC). Questionnaires on characteristics and Malaysian versions of Oral Health Impact Profile (OHIP-14(M)) and Health Assessment Questionnaire (HAQ-DI)) were answered.

**Results:**

A total of 187 subjects were included (29 RA-PD, 58 RA, 43 PD and 57 HC). OHIP-14(M) severity score was highest in the PD group (17.23 ± 10.36) but only significantly higher than the HC group (*p* < 0.05). The HAQ-DI scores of the RA group was significantly higher than the PD and HC groups (*p* < 0.05). The interaction between the effects of PD and RA on the OHRQoL and HRQoL was statistically significant (*p* < 0.05).

**Conclusion:**

PD and RA subjects both suffer impacts on their OHRQoL and HRQoL respectively. The interaction effect of both diseases significantly conferred impacts on their OHRQoL and HRQoL as measured by the OHIP-14(M) and HAQ-DI.

## Background

Periodontal disease has been identified by the World Health Organization (WHO) to be a significant contributor to the global burden of oral disease and is reported to be the 6^th^ most prevalent disease globally [1, 2]. Severe periodontitis (PD) has an overall worldwide prevalence of 11.2% [2]. On the other hand, rheumatoid arthritis (RA) has a significantly lower prevalence globally of 1% [3, 4]. Many epidemiological studies have concluded that there is a considerable positive association between these two diseases. While the relationship is unlikely to be causal, most of these studies have reported that PD is more severe and common in patients with established RA [5–7]. The plausible associations between PD and RA have been attributed to shared similar risk factors, tissue and bone destruction pathways, disease progression and immunogenetics [5].

There is a growing recognition that a true picture of a disease cannot be captured using traditional clinical measurement parameters alone but should be ideally supplemented by the individual’s point of view to give a more holistic representation [8]. This emphasis on quality of life (QoL) shows that the betterment of life holds just as much importance as the prolonging of it and rendering it disease-free [9]. Various instruments have been designed to gauge either the general well-being of the patient or more specific to the disease being investigated.

The most commonly used instrument for health related quality of life (HRQoL) in RA patients is the Health Assessment Questionnaire (HAQ) [10]. The HAQ centers on 5 dimensions, namely, ‘disability’, ‘pain’, ‘medication effects’, ‘costs of care’ and ‘mortality’ [10]. The “Short/2-page HAQ” which measures only the ‘disability’ dimension (HAQ-DI) and has now been cross-culturally adapted and translated into more than 60 different languages and dialects [11]. It was validated as the Malay version of the HAQ (Malay-HAQ) in 2008 for use among the RA population in Malaysia [12]. It can be administered in 5 min and scored within a minute which renders it a favourable instrument in population studies. One of the most widely used instruments to measure oral health-related QoL (OHRQoL) in patients with PD is the Oral Health Impact Profile (OHIP) [13]. This instrument has 49 questions which fall under seven dimensions or subscales, namely ‘functional limitation’, ‘physical pain’, ‘psychological discomfort’, ‘physical disability’, ‘psychological disability’, ‘social disability’ and ‘handicap’. A shorter version (OHIP-14) was created in 1997 for the ease of use [14]. In Malaysia, the OHIP-49 was shortened, translated and adapted for the Malaysian population by performing a thorough cross-cultural adaptation process and coined as the OHIP-14 (M) in 2005 [15].

It is recognized that RA has a deleterious effect on not only the physical, but also psychological and social functioning aspects of life [16]. Multiple studies using different instruments demonstrated that the detrimental effect of RA extends to the QoL of the subjects involved [16–19]. OHRQoL too has been demonstrated to be directly affected by PD [20–24] or RA [25, 26] respectively. Based on these findings it would be expected that patients suffering from concurrent RA and PD would have a similar or perhaps synergistic effect on their QoL. Currently there is no published study investigating the impact of those suffering from RA with PD on their HRQoL and OHRQoL. Hence the aim of this study was to assess the quality of life and its impacts on subjects with RA and PD**.**

## Methods

### Study design

This comparative cross-sectional study was conducted between November 2017 and December 2018. RA subjects were recruited from the Rheumatology Clinic in the University of Malaya Medical Centre (UMMC), Kuala Lumpur, Malaysia while the PD subjects and healthy controls were recruited from the Primary Care Unit, Faculty of Dentistry, University of Malaya, Kuala Lumpur, Malaysia.

The RA subjects had been diagnosed with RA based on the 2010 classification by the American College of Rheumatology and European League Against Rheumatism (ACR-EULAR) [27] with a duration of more than a year. They were subdivided into those with PD (RA-PD group) and those without PD (RA group). The non-RA subjects were divided into PD and healthy controls (HC). The presence or absence of PD was determined using the Centers for Disease Control and Prevention—American Academy of Periodontology (CDC-AAP) case definitions [28].

The inclusion criteria for the study was having at least 8 teeth excluding third molars. The exclusion criteria were subjects who had received either periodontal treatment or antibiotics during the preceding 4 months before the study; who had any concurrent systemic or debilitating conditions such as diabetes mellitus or other autoimmune diseases or who were pregnant. All subjects who satisfied the inclusion and exclusion criteria and provided written informed consent were enrolled into the study.

### Sample size calculation

Sample size was calculated using a study by Mulhberg and colleagues in 2017 as reference [29]. The mean (M) and standard deviation (SD) values for the German version OHIP-14 scores both RA and non-RA groups were M:7.7, SD:9.6 and M:1.6, SD:3.0 respectively. The sample size for this phase of study was calculated to be 35 subjects for all 4 groups using the PS (Power and Sample Size Calculation) software (Vanderbilt University) version 3.0.43.

### Measurements

Questionnaires were completed by all participants before clinical examinations were done. The first section of the questionnaire consisted of questions pertaining to social demographics, medical and dental history. The second section was the OHIP-14(M) questionnaire [15]. Subjects were required to report on the frequency of experiencing negative impacts over a 1-year period affecting seven domains. The third section was on the HAQ-DI [12]. Based on a 20-question questionnaire, subjects were required to report disability over the last 1-week period. “Background” section was administered by the examiner whereas “Methods” and “Results” sections were self-administered. A pre-test of the questionnaire was performed on 10 subjects from the Faculty of Dentistry, University of Malaya in August 2017 prior to subject recruitment for face validity.

All subjects were then subjected to a full mouth periodontal examination (excluding 8′s) comprising pocket probing depth (PPD) and gingival recession (GR) on 6 sites (mesio-buccal, mid-buccal, disto-buccal, mesio-lingual/palatal, mid-lingual/palatal, disto-lingual/palatal) on each tooth and the clinical attachment level (CAL) was thus obtained. Visible Plaque Index (VPI) [30] and Gingival Bleeding Index (GBI) [30] were recorded at 4 sites per tooth (mesio-buccal, mid-buccal, disto-buccal and palatal/lingual). Total number of teeth present was also recorded. A UNC 15 periodontal probe (Hu Friedy®, Chicago, IL, USA) was used for all periodontal clinical examination. All clinical assessments were done by three calibrated examiners. Kappa scores of more than 0.75 was obtained by all 3 examiners for both intra-examiner and inter-examiner standardizations of PPD and CAL and thus were considered “reproducible” and “standardized”.

The PD status of the subjects was classified according to the CDC-AAP case definitions whereby PD subjects had to have ≥ 2 interproximal sites with CAL ≥ 3 mm, and ≥ 2 interproximal sites with PD ≥ 4 mm (not on the same tooth) or one site with PD ≥ 5 mm [28]. The duration of RA disease of all RA subjects were obtained from the patient software registry of the University of Malaya Medical Centre (UMMC).

The research was conducted in full accordance with the World Medical Association’s Declaration of Helsinki. Ethical approval was obtained from the Medical Research Ethics Committee (MREC), University of Malaya Medical Centre (UMMC) (Reference number: MRECID.NO: 2017510-5227) and the Medical Ethics Committee, Faculty of Dentistry, University of Malaya (Reference number: DF RD1707/0029(L)).

### Data analyses

Two parameters of OHIP-14 (M) were computed—the prevalence and severity of impacts. The prevalence was defined as the percentage of people reporting one or more items ‘quite often’ or ‘very often’. The severity of impacts was the sum of the ordinal responses on the 5-point Likert scale for all 14 questions whereby the higher the score, the poorer the OHRQoL of the subject. If more than 20% of the items were coded missing, then the participant was excluded from further analysis. Otherwise, the values were imputed using the mean value of that particular item. Similar procedures were done for items with a “don’t know” response. Hence possible scores ranged from 0 to 56. On the other hand, for the HAQ-DI, severity scores were calculated by choosing the greatest score (0–3) from each part within the eight categories. These 8 highest scores for their respective categories were then averaged out to get a final mean which has 25 possible values from 0 to 3.

The differences between groups of subjects (divided into categorical and continuous data) were analyzed using the Pearson Chi Square-test and Anova or Kruskal–Wallis tests respectively. Two-way Anova was performed to confirm the interaction effect of both RA and PD status on the HRQoL and OHRQoL. Multiple linear regression analysis was performed to analyze the relationship between age, gender, education level and brushing frequency with the OHRQoL of subjects. Data analysis was performed using the Statistical Package of Social Sciences (SPSS) (SPSS, Chicago, IL, USA) Version 23.0.

## Results

The sample characteristics of all 4 groups of subjects are demonstrated in Table 1. There were 29 RA-PD subjects, 58 RA subjects, 43 PD subjects and 57 HCs. Most of the subjects recruited were females. The RA-PD group had the highest mean age at 55 ± 9.3 whereas the HC group had the lowest mean age at 32.1 ± 12.8. The majority of the subjects in all 4 groups had education up to secondary and tertiary levels. There was a significant difference (*p* < 0.01) between groups in terms of gender, mean age and education level. Subjects with Chinese descent made up the largest majority of all groups (44.2–56.9%) except in the PD group which had a larger Malay race proportion at 47.4%. A large majority of the subjects in all groups never smoked (83.7–100%).

**Table 1:** Sample characteristics of subjects of all groups

Sample characteristics	RA-PD (n = 29)	RA (n = 58)	PD (n = 43)	HC (n = 57)	*p* value^a^
Gender, n (%)					
Male	7 (24.1)	6 (10.3)	20 (46.5)	20 (35.1)	< 0.01*
Female	22 (75.9)	52 (89.7)	23 (53.5)	37 (64.9)	
Age group, n (%)					
Below 30	0 (0)	1 (1.7)	13 (30.2)	30 (52.6)	< 0.01*
30–44	5 (17.2)	11 (19.0)	15 (34.9)	18 (31.6)	
45 and above	24 (82.8)	46 (79.3)	15 (34.9)	9 (15.8)	
Mean Age (Mean ± SD)	55.0 ± 9.3	54.7 ± 10.6	40.0 ± 15.0	32.1 ± 12.8	< 0.01^b^*
Ethnicity, n (%)					
Malay	9 (31.0)	13 (22.4)	18 (41.9)	27 (47.4)	0.191
Chinese	13 (44.8)	33 (56.9)	19 (44.2)	20 (35.1)	
Indian	6 (20.7)	12 (20.7)	5 (11.6)	8 (14.0)	
Others	1 (3.5)	0 (0)	1 (2.3)	2 (3.5)	
Education, n (%)					
Primary	1 (3.4)	3 (5.2)	0 (0)	0 (0)	< 0.01*
Secondary	20 (69.0)	23 (39.7)	14 (32.6)	8 (14.0)	
Tertiary	8 (27.6)	32 (55.2)	29 (67.4)	49 (86.0)	
Monthly Household Income (in Malaysian Ringgit)					
< 1999	5 (17.2)	9 (15.5)	12 (27.9)	15 (26.3)	0.561
2000–4999	9 (31.0)	26 (44.8)	20 (46.5)	24 (42.1)	
5000–9999	14 (48.3)	21 (36.2)	10 (23.3)	17 (29.8)	
> 10,000	1 (3.4)	2 (3.4)	1 (2.3)	1 (1.8)	
Smoking, n (%)					
Current smoker	2 (6.9)	0 (0)	4 (9.3)	5 (8.8)	0.078
Former smoker	2 (6.9)	0 (0)	3 (7.0)	1 (1.8)	
Non smoker	25 (86.2)	58 (100)	36 (83.7)	51 (89.5)	
Mean duration of RA diagnosis (Mean ± SD)	9.72 ± 9.30	10.67 ± 9.24	–	–	0.916

RA, subjects with rheumatoid arthritis only; PD, subjects with periodontitis only; RA-PD, subjects with RA and PD; HC, healthy controls

^*^Significant difference observed between groups at *p* < 0.05

^a^Pearson Chi-Square Test

^b^Kruskal–Wallis Test

The clinical periodontal parameters recorded for each group are shown in Table 2. The subjects in the HC group had significantly more teeth than the RA-PD and RA groups (*p* < 0.05). Both groups of subjects without PD showed significantly lower PPD, CAL and GBI scores than their counterparts in RA-PD and PD groups (*p* < 0.05). The VPI scores of both groups with PD were significantly higher than the HC group (*p* < 0.05). There was no difference in all periodontal parameters between RA-PD and PD groups.

**Table 2:** Clinical periodontal parameters of subjects of all groups

Clinical Periodontal Parameters	RA-PD (n = 29)	RA (n = 58)	PD (n = 43)	HC (n = 57)	*p* value
Number of teeth (mean ± SD)	24.10 ± 5.96^z^	25.33 ± 5.09^y^	27.63 ± 4.50	28.81 ± 2.39^zy^	< 0.01^b^*
PPD (mean ± SD)	2.93 ± 0.67^zy^	1.95 ± 0.29^zx^	3.29 ± 0.83^xw^	2.19 ± 0.99^yw^	< 0.01^b^*
CAL (mean ± SD)	3.61 ± 1.09^zy^	0.72 ± 0.23^zx^	4.36 ± 3.12^xw^	0.65 ± 0.19^yw^	< 0.01^b^*
VPI in % (mean ± SD)	51.25 ± 29.13^z^	40.90 ± 27.10^y^	54.84 ± 26.89^yx^	29.90 ± 23.43^zx^	< 0.01^a^*
GBI in % (mean ± SD)	28.13 ± 21.61^zy^	7.51 ± 7.67^zx^	30.50 ± 22.95^xw^	9.25 ± 11.43^yw^	< 0.01^b^*

RA, subjects with rheumatoid arthritis only; PD, subjects with periodontitis only; RA-PD: subjects with RA and PD; HC: healthy controls; PPD: probing pocket depth; CAL: clinical attachment loss; VPI, visible plaque index; GBI: gingiva bleeding index

*Statistically significant between 2 or more groups at *p* < 0.05; w, x, y, z: Statistically significant difference between 2 groups at *p* < 0.05 (Tukey HSD & Dunnet T3)

^a^One-way Anova Test

^b^Kruskal–Wallis Test

Table 3 shows the prevalence and severity impacts of the OHRQoL of all 4 groups of subjects. On a subject level, the highest prevalence of impact on the OHRQoL was reported to be 69.8% in the PD group followed by the HC, RA and RA-PD groups at 65.1%, 62.1% and 58.6% respectively. The differences between groups were however not statistically significant (*p* > 0.05).

**Table 3:** The prevalence and severity of impacts by dimensions, items and overall OHIP-14 (M) scores between all groups

Dimensions and items	OHIP-14 (M) Scores									
	Prevalence: n (%)				*p* value^a^	Severity: mean ± SD				*p* value
	RA-PD (n = 29)	RA (n = 58)	PD (n = 43)	HC (n = 57)		RA-PD (n = 29)	RA (n = 58)	PD (n = 43)	HC (n = 57)	
Functional limitation	9 (31.3)	8 (13.8)	12 (27.9)	10 (17.5)	0.213	2.93 ± 1.91^z^	2.07 ± 1.80	3.00 ± 2.26^y^	1.77 ± 1.71^zy^	0.003^b^*
Difficulty in chewing	4 (13.8)	5 (8.6)	9 (20.9)	2 (3.5)		1.45 ± 1.27	1.09 ± 1.08	1.47 ± 1.37	0.82 ± 0.95	
Bad breath	6 (20.7)	4 (6.9)	9 (20.9)	8 (14.0)		1.48 ± 1.35	0.98 ± 1.12	1.53 ± 1.20	0.95 ± 1.23	
Physical pain	4 (13.8)	12 (20.7)	12 (27.9)	9 (15.8)	0.238	2.21 ± 1.40	2.47 ± 1.82	2.70 ± 1.75	2.18 ± 1.67	0.431^b^
Discomfort eating	3 (10.3)	10 (17.2)	10 (23.3)	4 (7.0)		1.07 ± 1.16	1.33 ± 1.32	1.56 ± 1.26	0.95 ± 1.04	
Oral ulcer	1 (3.4)	5 (8.6)	3 (7.0)	7 (12.3)		1.14 ± 0.92	1.14 ± 1.07	1.14 ± 0.99	1.23 ± 1.00	
Psychological discomfort	13 (44.8)	32 (55.2)	24 (55.8)	21 (36.8)	0.213	3.10 ± 1.74	3.36 ± 1.87	3.86 ± 2.13	3.04 ± 2.01	0.187^b^
Discomfort due to food stuck	12 (41.4)	29 (50.0)	22 (51.1)	19 (33.3)		2.31 ± 1.28	2.41 ± 1.16	2.30 ± 1.10	1.86 ± 1.03	
Felt shy	1 (3.4)	8 (13.8)	9 (20.9)	9 (15.8)		0.79 ± 0.90	0.95 ± 1.23	1.56 ± 1.35	1.18 ± 1.30	
Physical disability	4 (13.8)	13 (22.4)	13 (30.2)	10 (17.5)	0.238	1.45 ± 1.74^z^	1.67 ± 2.07^y^	2.67 ± 1.97^zy^	1.77 ± 1.75	0.023^b^*
Avoid eating food	2 (6.9)	11 (19.0)	11 (25.6)	4 (7.0)		0.97 ± 1.02 0.48 ± 1.02	1.16 ± 1.30	1.67 ± 1.25	0.88 ± 1.04	
Avoid smiling	2 (6.9)	5 (8.6)	4 (9.3)	8 (14.0)			0.52 ± 1.06	1.00 + 1.25	0.89 ± 1.09	
Psychological disability	1 (3.4)	2 (3.4)	5 (11.6)	6 (10.5)	0.213	0.76 ± 1.24	1.12 ± 1.46	1.84 ± 2.06	1.53 ± 1.96	0.085^c^
Disturbed sleep	0 (0)	1 (1.7)	2 (4.7)	4 (7.0)		0.24 ± 0.58	0.47 ± 0.75	0.84 ± 1.07	0.65 ± 1.01	
Disturbed concentration	1 (3.4)	2 (3.4)	5 (11.6)	5 (8.8)		0.52 ± 0.83	0.66 ± 0.95	1.00 ± 1.18	0.88 ± 1.09	
Social disability	0 (0)	1 (1.7)	1 (2.3)	1 (1.8)	0.261	0.41 ± 0.91	0.53 ± 1.17	0.98 ± 1.50	0.53 ± 1.05	0.313^c^
Avoid going out	0 (0)	1 (1.7)	0 (0)	0 (0)		0.10 ± 0.41	0.22 ± 0.62	0.30 ± 0.67	0.18 ± 0.47	
Daily activities disturbed	0 (0)	1 (1.7)	1 (2.3)	1 (1.8)		0.31 ± 0.66	0.31 ± 0.65	0.67 ± 0.99	0.35 ± 0.67	
Handicap	1 (3.4)	8 (13.8)	11 (25.6)	6 (10.5)	0.213	0.86 ± 1.06^zy^	1.84 ± 1.99^z^	2.19 ± 1.89^y^	1.33 ± 1.62	0.012^c^*
Spending money	1 (3.4)	6 (10.3)	5 (11.6)	2 (3.5)		0.45 ± 0.74	1.26 ± 1.22	1.02 ± 1.14	0.58 ± 0.91	
Less confident	0 (0)	5 (8.6)	7 (16.3)	5 (8.8)		0.41 ± 0.73	0.59 ± 1.03	1.16 ± 1.34	0.75 ± 1.02	
OHIP-14 (M)	17 (58.6)	36 (62.1)	30 (69.8)	28 (65.1)	0.213	11.72 ± 7.18	13.23 ± 7.89	17.23 ± 10.36^z^	12.14 ± 9.59^z^	0.020^b^*

OHIP-14 (M), Oral Health Impact Profile Shortened Malaysian Version; RA, subjects with rheumatoid arthritis only; PD, subjects with periodontitis only; RA-PD, subjects with RA and PD; HC, healthy controls

*Statistically significant difference between two of more groups at *p* < 0.05; z, y: Statistically significant difference between two groups at *p* < 0.05 (Tukey HSD)

^a^Pearson Chi-Square Test

^b^One-way Anova Test

^c^Kruskal-Wallis Test

The severity of OHIP-14 (M) scores was the highest in the PD group (17.23 ± 10.36) but was only significantly higher than the HC group (12.14 ± 9.59). The RA group reported higher severity OHIP-14 (M) scores than the RA-PD group (13.23 ± 7.89 vs 11.72 ± 7.18) but the difference was not statistically significant (*p* > 0.05).

The severity of impacts on the dimensions of ‘physical pain’, ‘psychological discomfort’, ‘psychological disability’ and ‘social disability’ were not significant between the 4 groups. However, there were statistically significant differences between groups in the dimensions of ‘functional limitation’, ‘physical disability’ and ‘handicap’ (*p* < 0.05). The severity scores of the HC group was significantly lower (*p* < 0.05) as compared to RA-PD and PD groups in the dimension of ‘functional limitation’. On the other hand, the PD group showed significantly higher severity scores in the dimension of ‘physical disability’ (the two items investigated are ‘avoiding eating’ and ‘avoiding smiling’) compared to the RA-PD and RA groups (*p* < 0.05). In the dimension of ‘handicap’ (the two items investigated were ‘spending money’ and being ‘less confident’), the RA-PD group reported a significantly lower severity score when compared to the RA and PD groups respectively (*p* < 0.05). The OHRQoL score of the PD group remained statistically significantly higher than the HC group even after multiple linear regression analysis to control for confounding factors (*p* = 0.01).

The HAQ-DI scores are shown in Table 4. On the subject level, the severity HAQ-DI score was highest in the RA group (0.85 ± 0.83) followed by the RA-PD (0.54 ± 0.49), PD (0.09 ± 0.15) and HC (0.08 ± 0.19) groups. The scores of the RA and RA-PD groups were significantly higher than the non-RA groups (*p* < 0.05) but not significantly different from each other (*p* > 0.05). However, after performing multiple linear regression analysis to control for confounding variables, the HRQoL score of the RA group remained significantly higher than both non-RA groups (*p* < 0.01) but the HRQoL of the RA-PD group was no longer significantly higher than the HC group (*p* > 0.05).

**Table 4:** The severity of impacts by dimensions and overall HAQ-DI scores between all groups

Disability categories	HAQ-DI Scores				
	Severity: (mean ± SD)				*p* value^a^
	RA-PD (n = 29)	RA (n = 58)	PD (n = 43)	HC (n = 57)	
Dressing and grooming	0.48 ± 0.57^zy^	0.64 ± 0.85^xw^	0.12 ± 0.39^zx^	0.04 ± 0.19^yw^	< 0.01*
Arising	048 ± 0.51^zy^	0.69 ± 0.80^xw^	0.09 ± 0.29^zx^	0.07 ± 0.26^yw^	< 0.01*
Eating	0.59 ± 0.63^zy^	1.02 ± 1.03^xw^	0.12 ± 0.32^zx^	0.04 ± 0.19^yw^	< 0.01*
Walking	0.48 ± 0.57^zy^	0.83 ± 0.92^xw^	0.09 ± 0.29^zx^	0.09 ± 0.29^yw^	< 0.01*
Hygiene	0.38 ± 0.62^z^	0.72 ± 0.85^yx^	0.02 ± 0.15^zy^	0.11 ± 0.31^x^	< 0.01*
Reach	0.69 ± 0.76^zy^	0.98 ± 1.16^xw^	0.16 ± 0.53^zx^	0.11 ± 0.31^yw^	< 0.01*
Grip	0.62 ± 0.68^zy^	0.98 ± 1.64^xw^	0.05 ± 0.21^zx^	0.11 ± 0.31^yw^	< 0.01*
Daily activities	0.62 ± 0.78^zy^	0.95 ± 1.08^xw^	0.09 ± 0.29^zx^	0.09 ± 0.29^yw^	< 0.01*
HAQ-DI	0.54 ± 0.49^zy^	0.85 ± 0.83^xw^	0.09 ± 0.15^zx^	0.08 ± 0.19^yw^	< 0.01*

HAQ-DI, Health Assessment Questionnaire Disability Index; RA, subjects with rheumatoid arthritis only; PD, subjects with periodontitis only; RA-PD, subjects with RA and PD; HC, healthy controls

*Statistically significant difference between 2 or more groups at *p* < 0.05; w, x, y, z: Statistically significant difference between 2 groups at *p* < 0.05 (Dunnet T3)

^a^Kruskal-Wallis Test

The interaction effect of both RA and PD on the OHRQoL and HRQoL of subjects is shown in Table 5. There was a statistically significant interaction between the effects of both RA and PD on the OHRQoL scores (F = 5.6, *p* < 0.05) and HRQoL scores (F = 4.2, *p* < 0.05).

**Table 5:** The effects of RA, PD and combined RA and PD on the overall OHIP-14(M) and HAQ-DI scores of all subjects (N = 187)

	OHIP-14 (M) Scores		HAQ-DI Scores	
	F	*p* value^d^	F	*p* value^a^
RA Status	2.8	0.094	60.9	0.000*
PD Status	1.9	0.170	3.5	0.062
Combined RA & PD Status	5.6	0.019*	4.2	0.041*

OHIP-14 (M), Oral Health Impact Profile Shortened Malaysian Version; HAQ-DI, Health Assessment Questionnaire Disability Index; RA, rheumatoid arthritis; PD, periodontitis

*Statistically significant at *p* < 0.05

^a^2-way Anova

## Discussion

When comparing the OHIP-14 (M) scores among groups, it was detected that the OHIP-14 (M) scores were significantly higher in the PD group as compared to the HC group. This is consistent with many studies that have reported poorer OHRQoL in PD subjects compared to their healthy counterparts [21, 23, 31, 32]. Other concerns such as dental caries, orthodontic malocclusions, endodontic conditions among others would also affect the OHRQoL of the subjects. However, within the limits of this study, the OHIP-14(M) appears to be sufficiently specific to report on the difference in OHRQoL between subjects with and without PD.

The OHRQoL of the PD group in this study was higher than that of the RA-PD group. Although not statistically significant (*p* > 0.05), it may be clinically relevant to the subjects individually. A possible explanation is that these 2 groups of PD patients have different motivations when first encountered. The PD subjects were recruited from the Primary Care Unit while the RA subjects were recruited from the Rheumatology Clinic. Similar to Needleman and colleagues’ study, this group of PD subjects represent a group which sought specialist periodontal care or were referred for it [22] whereas all 29 RA subjects who were diagnosed with PD were not aware of their oral and periodontal condition prior to their recruitment in the study. The self-awareness of an undiagnosed or untreated PD condition (a silent disease in nature) might result in the self-reporting of poorer OHRQoL among the PD group. Similarly, this might also explain why the PD group demonstrated the highest prevalence of ‘quite often’ and ‘very often’ reporting in all dimensions of the OHIP-14 (M) compared to the other 3 groups.

In this study, the OHRQoL of both the RA groups were not significantly different (*p* > 0.05) despite the differences in their periodontal status. There is no current literature that has compared the impact of PD on the OHRQoL of RA subjects hence we are not able to draw any comparisons at this point of time. However, similar findings have been reported in studies comparing the impact of PD on subjects with other systemic diseases. In their 2015 study in the UK, Irani et al. concluded that the OHRQoL of subjects with Type II Diabetes Mellitus (T2DM) was not significantly different regardless of their PD status. However, it was lower than that of the subjects with PD but without T2DM [33]. As in the case with the T2DM subjects, the lack of impact of PD on the OHRQoL of RA-PD subjects might be due to the burden of the chronic nature of RA on the individual, hence minimizing the impact on the oral health related dimensions within the OHIP-14(M).

Our study is the first to compare the impact of PD on the HRQoL of RA and non-RA patients using the HAQ. There was no significant difference in the HRQoL between the RA and RA-PD groups. After regression analysis, the HRQoL scores of the RA group was significantly higher (*p* < 0.01) than that of the non-RA groups. This indicates that the subjects in the RA group had significantly poorer HRQoL compared to their non-RA counterparts. The finding is in agreement with that reported by both Haroon et al. and Husted et al. [34, 35] that RA subjects have poorer HRQoL than their healthy counterparts. However, the regression analysis also showed that the HRQoL scores of the RA-PD group was significantly higher than the PD group (*p* < 0.01) but not significantly higher than the HC group (*p* = 0.07). This indicated that after controlling for all confounding factors, the HRQoL of the RA-PD group was not significantly worse than the HC group. As with what was reported by Irani et al. regarding OHRQoL in T2DM subjects [33], a similar pattern is seen whereby the lack of impacts reported in the HAQ-DI by RA-PD subjects might be due to the chronic nature of PD leading to an increase in the tolerance to the physical burdens of their underlying RA disease. Hence, minimizing the impact on the health related dimensions within the HAQ-DI in these subjects. In addition to this, the HC group met the inclusion criterion of “no other systemic disease” but might still be experiencing an ailment which impacts the HRQoL as measured by the HAQ-DI. A post-hoc calculation indicated sufficient power (99.8%) despite the disparity in sample sizes between both these groups of subjects (RA-PD vs HC groups).

When we assessed the entire sample population of 187 subjects, we found that the interaction between the effects of both RA and PD diseases on the OHRQoL and HRQoL were statistically significant (*p* < 0.05). Systematic review has shown that the multiple morbidities have a negative influence on QoL, especially HRQoL [36]. The HAQ-DI and the OHIP-14(M) are both specific instruments for the measurement of QoL of rheumatic diseases and oral diseases respectively. Hence these instruments may not be sufficient to detect the synergism of impacts by PD and RA on the HRQoL of subjects. The HAQ-DI investigates only impacts in the physical disability dimension over a period of a week which might be sensitive enough to capture the impacts of RA but not necessarily PD. On the other hand, while the OHIP-14(M) captures the impacts in a wider range of dimensions, it might fail to identify impacts of RA. This might be because the questions are from an oral health perspective. However, by running the 2-way Anova, we managed to investigate and draw more meaningful conclusions regarding the interaction between the effects of RA and PD on the QoL of the subjects involved.

The strength of this study is it being the first to investigate the interaction effect of PD in RA patients and the impact on their quality of life in the Malaysian population. The findings give a new insight into the association between diseases and the impacts on the QoL of those suffering from it. Although the prevalence of RA subjects globally is low compared to the prevalence of PD, the implications of PD in RA patients are important. Hence, understanding the nature of association and patient-centered outcomes is crucial. Another strength of this study is that we included control groups for both RA and PD cases. This enabled meaningful comparisons to be made. Additionally, the present study’s strict inclusion criteria enabled the collection of a higher quality pool of subjects who better reflect their populations.

There were some limitations to our study. Due to the strict inclusion/exclusion criteria and the limited number of patients available in the Rheumatology clinic we were unable to achieve the required sample size of 35 in the RA-PD group and this may have an effect on our findings. Our study however included a sufficiently large sample size in the other groups as compared to certain studies [4, 37] and comparable to other studies [38, 39]. Another limitation of the study was that the groups were not balanced in the distribution of gender, age and education. To overcome this bias, regression analysis was performed. Another limitation is that there was heterogeneity among the RA subjects with regards to the medications and current disease activity. It is unclear how the medications taken might affect their QoL.

The clinical relevance of this study is however undeniable. It gives us a better insight about the patient-centered outcomes reported by patients suffering from PD, RA or both diseases. This extra paradigm of knowledge enables clinicians to better empathize and customize management strategies to not just manage the disease but also address the dimensions of needs.

In view of the findings of this study, together with the stated limitations, we recommend that future studies should include more centers (hospitals and RA support groups) to better capture the general RA population and investigate the DAS-28 scores of RA subjects to get a clearer picture of their present disease status.

## Conclusions

Within the limits of this study, the following conclusions can be drawn. PD and RA subjects both suffer impacts on their OHRQoL and HRQoL respectively. The interaction effect of both diseases significantly conferred impacts on their OHRQoL and HRQoL as measured by the OHIP-14(M) and HAQ-DI.

## Data Availability

The datasets used and/or analysed during the current study are available from the corresponding author on reasonable request.

## Abbreviations

ACR/EULAR: American College of Rheumatology/European League Against Rheumatism
BOP: Bleeding on probing
CAL: Clinical attachment loss
CDC-AAP: Centers for Disease Control and Prevention—American Academy of Periodontology
CEJ: Cemento-enamel junction
CI: Confidence interval
DAS: Disease activity score
GBI: Gingival Bleeding Index
GR: Gingiva recession
HAQ: Health Assessment Questionnaire
HAQ-DI: Health Assessment Questionnaire-Disability Index
HC: Subjects without RA and PD/healthy controls
HRQoL: Health Related Quality of Life
OHIP: Oral Health Impact Profile
OHIP-14 (M): Malaysian Version of OHIP-14
OHRQoL: Oral Health Related Quality of Life
PD: Periodontitis
PPD: Probing pocket depth
QoL: Quality of life
RA: Rheumatoid arthritis
RA-PD: Subjects with RA and PD
SD: Standard deviation
SPSS: Statistical Package of Social Sciences
T2DM: Type II Diabetes Mellitus
UMMC: University of Malaya Medical Centre
VPI: Visible Plaque Index
WHO: World Health Organisation
